# Implication on the prognosis according to the cause of heart failure decompensation

**DOI:** 10.1186/cc14665

**Published:** 2015-09-28

**Authors:** Pedro Gabriel MB Silva, Flavio Brito, Mariana Okada, Patricia Roveri, Douglas Ribeiro, Giuliano Generoso, Jose Teixeira, Thiago Macedo, Antonio Baruzzi, Valter Furlan

**Affiliations:** 1 Hospital Totalcor, Cerqueira Cesar, São Paulo, São Paulo, Brazil

## Introduction

Heart failure (HF) is responsible for the majority of hospitalizations due to cardiovascular disease, and different clinical triggers are related to the cardiac decompensation.

## Objective

To evaluate the prognosis of patients hospitalized due to acute HF, according to the cause of decompensation.

## Methods

We retrospectively evaluated data from 731 patients consecutively admitted to a private cardiovascular center due to acute HF during 2013. We analyzed the frequency of each factor assigned as the trigger for the decompensation of HF among these patients, and also the length of stay and the number of deaths in each group. The infection group was compared with the other two groups separately, using Fisher's exact test for categorical variables and Student's *t *test for continuous variables.

## Results

The factor "infection" was associated with more days of hospitalization (Table 1), above the average of other triggers (10 × 6.95 days; p <0.01). The number of days in ICU in the cases of decompensation due to infection was also higher than the average from other causes (5.8 × 3.35 days; p <0.01). In addition, of the 48 deaths in 2013, 58% (n = 28) were in patients with decompensated HF due to infection, and among these 28 deaths 15 were secondary to evolution of sepsis, in 6 there were predominance of the cardiac condition while the remaining 7 deaths showed mixed shock (cardiac and septic) or other complications related to both conditions leading to death. Conclusion: Infection was the main factor of decompensation, requiring a longer hospital stays, more days in the ICU and being responsible for most of the deaths occurred in patients hospitalized for acute HF. Studies of specific approaches in acute HF triggered by infection are warranted.

**Table 1:** 

	Infection (*n *= 253)	Noncompliance (*n *= 126)	Progress of disease (*n *= 191)	*p *value
ICU stay (days)	5.8 (± 9)	3.7 (± 3.9)	3.2 (± 3.1)	0.013 and <0.001
Length of stay (days)	10 (±9.2)	7 (± 5.7)	8.1 (± 7.3)	0.019 and <0.001
In-hospital mortality (%)	11.1	3	5.2	0.009 and 0.039
Readmission in 30 days (%)	21.3	19.8 %	12	0.78 and 0.011

## Conclusion

Infection was the main factor of decompensation, requiring a longer hospital stay, more days in the ICU and being responsible for most of the deaths occurring in patients hospitalized for acute HF. Studies of specific approaches in acute HF triggered by infection are warranted.

